# Factors Influencing the Improvement of Activities of Daily Living during Inpatient Rehabilitation in Newly Diagnosed Patients with Glioblastoma Multiforme

**DOI:** 10.3390/jcm11020417

**Published:** 2022-01-14

**Authors:** Keisuke Natsume, Harutoshi Sakakima, Kentaro Kawamura, Akira Yoshida, Shintaro Akihiro, Hajime Yonezawa, Koji Yoshimoto, Megumi Shimodozono

**Affiliations:** 1 Division of Rehabilitation, Kagoshima University Hospital, 8-35-1 Sakuragaoka, Kagoshima 890-8520, Japan; mx2015@m2.kufm.kagoshima-u.ac.jp (K.N.); kentarok@m.kufm.kagoshima-u.ac.jp (K.K.); akiray@m.kufm.kagoshima-u.ac.jp (A.Y.); akihirot@m2.kufm.kagoshima-u.ac.jp (S.A.); rihakoza@m2.kufm.kagoshima-u.ac.jp (M.S.); 2 Department of Physical Therapy, School of Health Sciences, Faculty of Medicine, Kagoshima University, Kagoshima 890-8544, Japan; 3 Department of Neurosurgery, Kagoshima University Graduate School of Medical and Dental Sciences, Kagoshima 890-8544, Japan; hajime@m3.kufm.kagoshima-u.ac.jp (H.Y.); kyoshimo@m.kufm.kagoshima-u.ac.jp (K.Y.)

**Keywords:** glioblastoma, performance status, motor paralysis, rehabilitation, overall survival

## Abstract

Glioblastoma multiforme (GBM) is the most common and aggressive brain tumor. To identify the factors influencing the improvement of the activities of daily living (ADL) in newly diagnosed patients with GBM, we investigated the characteristics and variable factors and overall survival. A total of 105 patients with GBM were retrospectively analyzed and categorized into the following three groups according to the quartile of change of their Barthel index score from admission to discharge: deterioration (*n* = 25), no remarkable change (*n* = 55), and good recovery (*n* = 25). A statistical difference was observed in the pre-operative, intra-operative, post-operative, and rehabilitation-related factors between the deterioration and good recovery groups. Multiple regression analysis identified the following significant factors that may influence the improvement of ADL after surgery: the improvement of motor paralysis after surgery, mild fatigue during radio and chemotherapy, and length up to early walking training onset. The median overall survival was significantly different between the deterioration (10.6 months) and good recovery groups (18.9 months, *p* = 0.025). Our findings identified several factors that may be associated with post-operative functional improvement in patients with GBM. The inpatient rehabilitation during radio and chemotherapy may be encouraged without severe adverse events and can promote functional outcomes, which may contribute to the overall survival of newly diagnosed patients with GBM.

## 1. Introduction

Glioblastoma multiforme (GBM) is the most common and aggressive brain tumor, with a median age of 65 years at diagnosis [1], and is reportedly diagnosed in 12% of all patients with brain tumor patients [2]. Initial treatment consists of safe surgical resection followed by concurrent radiotherapy and temozolomide (TMZ) and another 6 months of adjuvant TMZ [3]. Gross total resection followed by radio and chemotherapy is suggested as optimal treatment, and radiotherapy and chemotherapy alone provide moderate clinical benefits [4]. The greater extent of resection at second surgery is associated with increased overall survival in patients with recurrent GBM [5]. Therapeutic advances in oncology have prolonged the survival of patients with brain tumors; however, some of these patients are often left with residual neurological deficits and psychological impairments [6]. However, patients with GBM experience long-term physical and psychological impairments that limit their daily activities because of factors related to the tumor or the treatment they receive [7,8,9].

Studies have demonstrated that the prognostic factors important for survival in patients with GBM are the age at surgery, location of tumor, nature of the tumor (multifocal or bilateral), and extent of resection [10,11]. The extent of resection at first and at recurrence is important predictor of outcome in patients with recurrent GBM [5]. The prognostic factor of functional outcome has been studied in patients with brain tumors using the functional assessment tool, performance status (PS) [12,13]. Notably, patient performance and function are usually assessed using the Karnofsky performance status (KPS), as a general assessment of patients with cancer [14]. In addition, preoperative PS has long been recognized as major independent prognostic factor in patients with GBM [13]. However, some patients with a good preoperative PS become functionally impaired during inpatient rehabilitation because of perioperative complications and surgically acquired neurologic deficits [13,15]. In contrast, some patients with poor preoperative PS show a good post-operative functional improvement after surgery [13]. Our anecdotal clinical experience seems to be associated with the pre-, intra-, and post-operative factors for the improvement of the patient’ functional status during inpatients rehabilitation with radio and chemotherapy. However, few studies have examined the pre-, intra-, and post-operative factors associated with the improvement of functional status, such as activities of daily living (ADL) in patients with GBM.

In addition, few investigations have been conducted to compare newly diagnosed patients with GBM who had aggravated or improved post-operative functional status during inpatient rehabilitation. To the best of our knowledge, one study reported a comparison between inpatient rehabilitation responders and non-responders based on the functional gains from admission to discharge using the functional independence measure [16]. In our hospital, almost all patients with GBM received rehabilitation services during radio and chemotherapy after surgery to improve neurological deficits or functional status, such as locomotion. In addition, we were assessed the amount of change in Barthel index (BI) scores from admission to discharge in patients with GBM, as an indicator of functional improvement. The BI score is correlated with the KPS of patients with brain tumors [17] and is easy to use for medical staff. Therefore, we compared newly diagnosed patients with GBM who had aggravated or improved activities of BI score, from admission to discharge, to examine the impact of inpatient rehabilitation during radio and chemotherapy on their functional improvement and survival.

Several studies have demonstrated that patients with brain tumors who received inpatient rehabilitation improved their functional status during the treatment course [6,7,12,16,18,19]. Exercise rehabilitation can maintain or improve functional performance and quality of life (QOL) in patients with GBM, even during medical treatment regimens [20]. In addition, clinical research has established the efficacy of appropriate exercises to counteract physical impairments, including fatigue and functional decline, cognitive impairment, and psychological effects such as depression and anxiety, in patients with brain tumors [21]. However, few studies examined the safety and effectiveness of inpatient rehabilitation intervention during radio and chemotherapy after tumor resection.

This study aimed to compare the characteristics, variable factors, and overall survival of patients with GBM who had aggravated or improved post-operative functional status during inpatient rehabilitation with radio and chemotherapy. In addition, we examined the pre-operative, intra-operative, post-operative, and rehabilitation-related factors associated with the improvement of post-operative ADL in newly diagnosed patients with GBM.

## 2. Methods

### 2.1. Study Design

A total 110 patients with newly diagnosed GBM, who were admitted to the neurosurgery department between January 2011 and October 2016, were analyzed in this study. All the patients underwent surgical tumor resection. Subsequently, they received rehabilitation services, including physical, occupational, and speech therapies during hospitalization for neurologic, physical, and psychological impairments. However, five patients did not receive inpatient rehabilitation after surgery. Therefore, a total of 105 patients with GBM were included in the study. This study retrospectively assessed the impact of impatient rehabilitation during radio and chemotherapy on the variable factors and overall survival of patients with deterioration and good recovery using the variation in BI scores from admission to discharge in newly diagnosed patients. This study was conducted in accordance with the Declaration of Helsinki and was approved by the ethical committee of our institution.

### 2.2. Patients Classification

**Figure 1:**
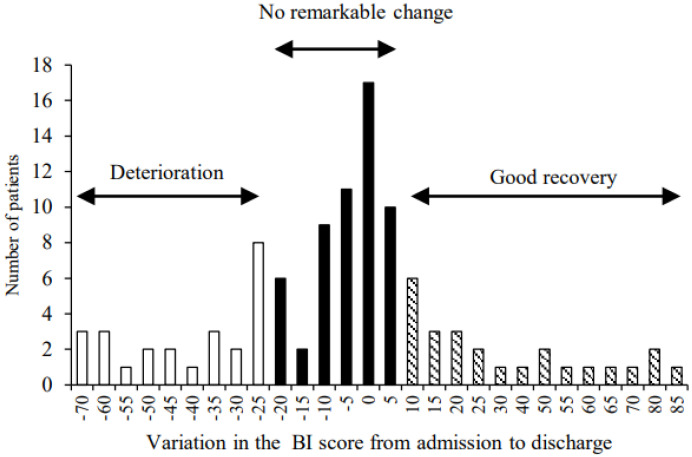
Category of three groups based on a quartile of variation of the Barthel index score: the 1st quartile (deterioration group, *n* = 25), the interquartile range (no remarkable change group, *n* = 55), and the 3rd quartile (good recovery group, *n* = 25).

We assessed the BI score of all patients at admission (pre-operation), 7 days after surgery, and at discharge. The median BI score of all patients was 65 (interquartile range (IQR): 35–90) at admission, 30 (IQR: 0–60) at 7 days after surgery, and 65 (IQR: 20–90) at discharge. Furthermore, we calculated the change in the BI score from admission to discharge, resulting in a median of BI score variation of −5 (IQR: −20–5). Therefore, the patients were categorized into the following three groups based on a quartile of variation of the BI score (Figure 1): the group of patients who experienced a deterioration in the BI score was placed under the 1st quartile (≤−25, *n* = 25, deterioration group) and the group of patients with improvement in the BI score was placed in the 3rd quartile (≥10, *n* = 25, good recovery group). The remaining patients were categorized into the group of patients with no remarkable change in the BI score and were placed in the IQR (*n* = 55, no remarkable change group).

### 2.3. Outcome Measures

Patient characteristics, such as age, sex, the KPS at admission, the extent of resection, the type of main treatment (TMZ, radiation, bevacizumab, TMZ concomitant radiation and bevacizumab), tumor location/hemisphere/size, the length of hospital stay, duration from initial symptoms to surgery, initial symptoms (motor paralysis and muscle weakness, cognitive dysfunction, headaches, visual, dysphagia, and fatigue), surgery details (surgery time, bleeding and transfusion volume, infusion volume, and fluid balance), adverse events during chemoradiotherapy, and duration from surgery to chemoradiotherapy were determined from the hospital’s medical records. The extent of resection was defined as gross and near total (the entire or >95% of the enhancing tumor was resected), partial (<95% tumor resection), and biopsy only.

With respect to post-operative events, we defined the presence or absence of fever (≥38 °C), infection, pneumonia, intracerebral hemorrhage, and motor paralysis of the lower limb that occurred 7 days after surgery. Furthermore, the rate of interruption and discontinuation of radio and chemotherapy was examined as a post-operative event. The level of motor paralysis was examined using the Brunnstrom recovery stage (BRS) of the lower limbs because the evaluation of the lower limbs was correlated with that of the upper limbs and hands. The deterioration of motor paralysis after surgery was defined as a decrease in ≥2 stages. In addition, we examined rehabilitation-related factors (length up to rehabilitation onset after surgery, length up to sitting and walking training onset, and patients with deterioration of motor paralysis and severe cognitive disorder or depression).

The severity of the adverse effects was evaluated according to the Common Terminology Criteria for Adverse Events (CTCAE) version 3.0 (grade 0: no adverse event; grade 1: mild; grade 2: moderate; grade 3: severe or medical intervention required; grade 4: life-threatening; grade 5: death). Hematology and other adverse events, including cognitive function, constipation, and fatigue, were assessed once per week during chemoradiotherapy. Severe cognitive impairment and depressive symptoms were defined as depression grade 1 or higher and severe cognitive impairment as grade 3 or higher.

Furthermore, we assessed the overall survival of patients with GBM. Overall survival was defined as the period from the surgery to the date of death or the date of the last follow-up for patients who were still alive. For all patients, the overall survival time was calculated in months.

### 2.4. Radio and Chemotherapy and Inpatient Rehabilitation

Standard radiation therapy was initiated within 2 weeks of tumor resection, and concomitant chemotherapy (TMZ) was initiated simultaneously. Subsequently, adjuvant TMZ was initiated 4 weeks after the end of radiotherapy for a period of 5 days every 28 days. Inpatient rehabilitation, including physical therapy, occupational therapy, and speech therapy, was initiated within 5 days after surgery in all patients. The rehabilitation intervention was performed for 40 min to 1 h per day for 5 days a week to improve functional impairment. Rehabilitation programs were provided by means of sitting, standing, and walking training as early as possible after resection based on the current trend of post-operative early mobilization in the intensive care unit [22,23]. Walking training was provided as an aerobic exercise with or without prosthetic devices, such as walkers or canes. In patients who faced difficulty walking, we attempted to mobilize the patients from the bed to a wheelchair to prevent disuse atrophy due to bed rest. However, it is difficult for some older patients to perform monotonous walking training owing to fatigue and mental stress caused by chemoradiotherapy. Therefore, the rehabilitation program was modified according to individual patient status.

### 2.5. Statistical Analysis

Statistical analyses were performed using the Shapiro–Wilk test followed by both parametric and non-parametric tests. Subsequently, the three groups were compared using either a one-way analysis of variance (ANOVA) or Kruskal–Wallis test, followed by Bonferroni’s post hoc tests for multiple comparisons. ANOVA was used to analyze age, tumor size, and the length of hospital stay. The Kruskal–Wallis test was used to examine the KPS at admission and BI score. Comparisons between the groups were performed using Student’s *t*-test or the Mann–Whitney U test. Student’s *t*-test was used to analyze the length from the initial symptoms to surgery, surgery time, bleeding volume, transfusion volume, infusion volume, fluid balance, length from surgery to chemoradiotherapy, length up to rehabilitation onset, length up to sitting, and walking training onset. Friedman’s test was used to examine the BI score and motor paralysis at admission, after surgery, and at discharge, followed by Bonferroni correction for multiple comparisons. The chi-square test or Fisher’s exact test was used for categorical variables. Cohen’s effect size was used to evaluate intergroup differences [24]. A stepwise multiple regression analysis was employed to determine the predictive factors associated with the variation in the BI score from admission to discharge. The dependent variables were adjusted for preoperative factors (age, the extent of resection, length from initial symptoms to surgery), intraoperative factors (fluid balance), post-operative factors (change in motor paralysis after surgery, fever, and fatigue), and rehabilitation factors (time to walking training initiation). As the data of 16 patients were missing, 89 patients’ data were used for the stepwise multiple regression analysis. The Kaplan–Meier overall survival time distributions were compared between the groups using the log-rank test. Follow-up of overall survival of seven patients was not possible because they treated another hospital in other prefecture (deterioration group; *n* = 3, no remarkable change group; *n* = 3, and good recovery group; *n* = 1). Therefore, overall survival of 98 patients was analyzed. The 95% confidence intervals (CIs) and corresponding *p* values were provided. Statistical significance was set at *p* < 0.05, and data are expressed as mean ± SD. Statistical analyses were performed using SPSS Statistics version 26 (IBM Corp., Armonk, NY, USA).

## 3. Results

### 3.1. Patient Characteristics

The clinical and tumor characteristics of the groups are presented in Table 1. The median KPS at admission in all patients was 70 or less, suggesting that, in the present study, patients showed a low PS before surgery. Notably, the KPS at admission in the good recovery group was significantly lower than that in the no remarkable change and deterioration groups (*p* < 0.01). Regarding the extent of resection, 62% of the patients underwent gross and near total resection. Approximately 86% of patients underwent radiation therapy with concomitant chemotherapy after surgery. There were no significant differences in the extent of resection, treatment, tumor location/hemisphere/size, and average hospital stay among the three groups. Interestingly, no patients with bilateral hemisphere tumors were observed in the good recovery group.

### 3.2. Change of ADL Level between Deterioration and Good Recovery Groups

Approximately 33% of all patients were improved more than preoperative ADL level at discharge, whereas 50% of all patients were worsened.

**Figure 2:**
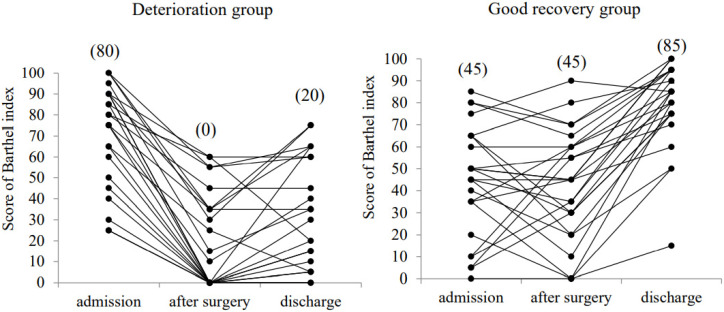
Change in the Barthel index score at admission, at 7 days after surgery (after surgery), and at discharge. The median values are shown in the parenthesis.

The changes in the individual BI scores during hospitalization in the deterioration and good recovery groups are shown in Figure 2. In the deterioration group, the mean BI score at admission (72.2 ± 23.8) was significantly decreased after surgery (18.4 ± 22.7, *p* < 0.01) and at discharge (30.6 ± 26.2, *p* < 0.01). In contrast, in the good recovery group, the mean BI score at admission (44.2 ± 25.0) was not significantly decreased after surgery (42.2 ± 25.0). The mean BI score was significantly improved at discharge (79.4 ± 19.3) compared with that at admission and after surgery (*p* < 0.01).

### 3.3. Comparison of the Deterioration and Good Recovery Groups in the Pre-Operative, Intra-Operative, Post-Operative, and Rehabilitation-Related Factors

The pre-operative, intra-operative, and post-operative factors in the deterioration and good recovery groups are shown in Table 2. Regarding the preoperative factors, the length from the initial symptoms to surgery was significantly longer in the deterioration group than in the good recovery group (*p* < 0.01), and there was a large intergroup effect size. Furthermore, the initial symptoms of motor paralysis and muscle weakness were significantly increased in patients in the good recovery group. These results suggest that patients in the good recovery group required early surgical intervention. Regarding the intraoperative factors, the fluid balance in the deterioration group was significantly increased compared to that in the good recovery group (*p* < 0.05) and showed a moderate intergroup effect size. Regarding post-operative factors, more than one type of symptom was recorded in both groups. Notably, motor paralysis after surgery was significantly worse in the deterioration group (36.0%) than in the good recovery group (4.0%, *p* < 0.01), which had a median intergroup effect size.

The rehabilitation-related factors for both groups are shown in Table 3. The deterioration group took a longer time to begin walking training than the good recovery group. In addition, seven patients (28.0%) in the deterioration group were unable to start ambulatory rehabilitation because of the exacerbation of the overall status. In contrast, all patients in the good recovery group were able to start walking training. With respect to motor paralysis, even though there was no significant difference in motor paralysis at admission between the groups, motor paralysis at discharge was significantly worse in the deterioration group than in the good recovery group (*p* < 0.01), which had a median intergroup effect size. The number of patients who did not show significant motor paralysis was larger in the good recovery group than in the deterioration group. Moreover, 50% of patients in the deterioration group exhibited worse motor paralysis from admission to discharge. Additionally, 40% of the patients in the deterioration group showed severe cognitive disorders or depression from admission to discharge. These factors were significantly different between the groups (*p* < 0.01), which had a high intergroup effect size.

The adverse events according to the CTCAE that occurred during chemoradiotherapy in both groups are shown in Table 4. There was no significant difference in hematologic toxicity between the groups. However, there was a significant difference in fatigue and fever between the groups (*p* < 0.01).

### 3.4. Improvement of ADL Level and Overall Survival

To identify the factors associated with the variation in BI scores during inpatient rehabilitation, we performed a stepwise multiple regression analysis. This analysis identified several significant factors: change in motor paralysis after surgery (β = 0.42, 95% CI: 6.5 to 13.7, *p* < 0.01), fatigue (β = −0.29, 95% CI: −23.1 to −7.0, *p* < 0.01), and the time to walking training onset after surgery (β = −0.28, 95% CI: −11.0 to −3.1, *p* < 0.01). Improvement of motor paralysis after surgery, mild fatigue during chemoradiotherapy, and time to early walking training onset were associated with a change in the BI score during inpatient rehabilitation.

**Figure 3:**
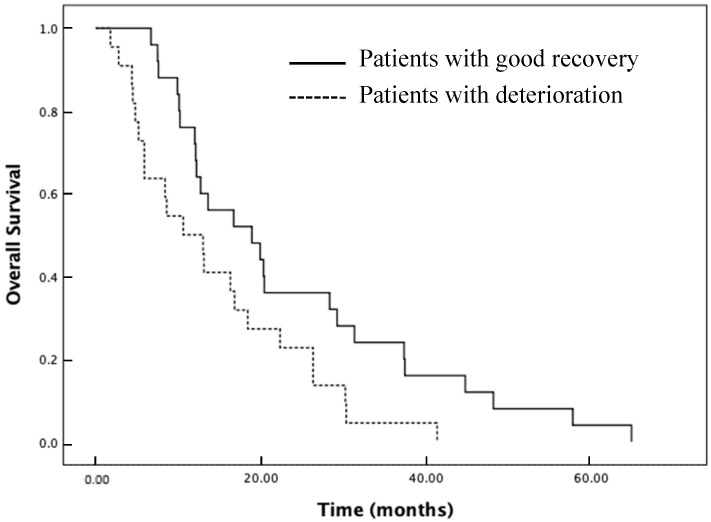
Kaplan–Meier estimates for mobility the deterioration (dotted line) and good recovery groups (solid line).

We further evaluated the differences in survival distributions between the deterioration and good recovery groups (Figure 3). The median overall survival for the entire cohort was 13.6 months (95% CI: 10.52 to 16.66). The median overall survival of the deterioration, no remarkable change, and good recovery group were 10.6 months (95% CI: 5.19 to 16.00), 13.4 months (95% CI: 8.04 to 18.75), and 18.9 months (95% CI: 8.61 to 29.18), respectively. The median overall survival was significantly longer in the good recovery group than in the deterioration group (Figure 3, *p* = 0.025).

## 4. Discussion

This study suggests that inpatient rehabilitation during radio and chemotherapy may be encouraged without severe adverse events. In addition, the patients in the deterioration group showed only a slight improvement from admission to discharge, despite their good preoperative BI score. In contrary, the BI score of the good recovery group significantly improved from admission to discharge, despite their poor preoperative BI score. Furthermore, our results suggested that the median overall survival was promoted by good functional recovery during early inpatient rehabilitation with radio and chemotherapy. Therefore, our findings suggest that even if patients with GBM show poor preoperative functional status, inpatient rehabilitation, as well as radio and chemotherapy, may be supported to improve the functional outcomes at discharge, which may contribute to the survival prognosis.

Various factors were associated with a good improvement in ADL during inpatient rehabilitation. Our findings suggest that the improvement of motor paralysis after surgery is the most important factor associated with good ADL recovery in patients with GBM. Motor deficits are one of the most important factors affecting the ability to perform ADL in stroke patients [25]. Therefore, the presence of motor paralysis as the initial symptom and its deterioration after surgery directly lead to functional disorders. Additionally, our results showed that mild fatigue and length up to early walking training onset after surgery, as well as the improvement of motor paralysis after surgery, were associated with a good improvement in ADL during early inpatient rehabilitation with radio and chemotherapy.

Fatigue is a common and severe symptom in patients with tumors, which often influences outcomes, such as functional status [26]. Additionally, severe fatigue correlates with poor physical function and QOL [27]. Chemotherapy-related fatigue peaks 1 day after chemotherapy, whereas radiation therapy-related fatigue gradually accumulates over the course of the treatment [28]. However, aerobic exercise has consistently been shown to alleviate cancer-related fatigue [27]. Furthermore, walking is an appropriate exercise for physical and mental disorders, such as disuse atrophy, depression, and anxiety. The present study suggests that early walking training may be important not only for the palliation of radio and chemotherapy-related fatigue but also for improving the functional or mental status of patients with GBM. Although rehabilitation interventions during radio and chemotherapy may not be popular in patients with GBM after surgery, the present study suggests that inpatient rehabilitation during radio and chemotherapy may be an important strategy to enhance the functional outcome of patients with GBM.

The factors that positively influence overall survival, except the preoperative KPS, are age at surgery, tumor location, and the extent of resection [11,13,29]. These tumor characteristics may be associated with the onset of post-operative motor paralysis and functional status. Preoperative factors including more than 75 years, frontal lobe tumor, overlapping lesion, size > 5.4 cm is associated with GBM related mortality [4]. However, our results did not show a statistical difference in the patient’s clinical and tumor characteristics, such as the age at surgery, tumor location, and the extent of resection in both groups. Gliomas extending into the insular or frontotemporal lobe are intricately related to functionally important structures, such as the motor fibers of the corona radiata and the internal capsule [30]. Furthermore, multifocal or bilateral tumors are important factors for survival in patients with GBM [11]. Therefore, tumor characteristics, including tumor location and hemisphere, may be associated with improvement in ADL. Further studies are needed to investigate the factors that influence the improvement of functional status during inpatient rehabilitation. Regarding intraoperative factors, early negative fluid balance is associated with low post-operative mortality in clinically ill patients following cardiovascular surgery [31]. Similarly, our results suggest that fluid balance may be associated with post-operative mortality following brain tumor resection. Therefore, the disorder of fluid balance may influence the functional status of patients with GBM after surgery.

Despite surgical resection and radiation therapy with or without adjuvant TMZ, the median overall survival of patients with GBM is less than 15 months [32]. A recent retrospective study reported that the 12-month and 3-year survival rates were 40% and 10%, respectively [33]. Our results showed that the median overall survival in the good recovery group was significantly longer than that in the deterioration group. Several studies have demonstrated that overall survival may be extended if rehabilitation intervention improves the physical function of patients with stroke and brain tumors [7,34]. Furthermore, significant improvements in the functional status and health-related QOL during inpatient rehabilitation have been associated with long survival after discharge [7,12]. Therefore, our results suggest that the overall survival of patients with GBM may be affected by improvements in functional status, including ADL ability, during early inpatient rehabilitation.

There was some limitation. First, this was a retrospective study with a relatively small sample size in a single hospital. Second, our participant differed in the preoperative functional status between the groups. The KPS was significantly different between the three groups. This study categorized the deterioration and good recovery groups using the variation in the BI score from admission to discharge, which may be influenced by the preoperative BI score. However, it is important for the patients to recover to the same or better than the preoperative ADL level. Therefore, this study designed to compare the patients with GBM who had aggravated or improved ADL from admission to discharge. Our results showed that approximately 50% of patients did not recover to preoperative ADL level. Therefore, continued rehabilitation intervention after radio and chemotherapy may be expected to improve their ADL level. Despite these limitations, the present study suggests that post-operative rehabilitation intervention during radio and chemotherapy can be performed without severe adverse events and can improve functional outcomes in patients with GBM.

In conclusion, our findings identified several factors that may be associated with post-operative functional improvement during inpatient rehabilitation during radio and chemotherapy, as follows: the improvement of motor paralysis after surgery, mild fatigue during radio and chemotherapy, and length up to early walking training onset. The inpatient rehabilitation during radio and chemotherapy may be encouraged without severe adverse events and can promote functional outcomes, which may contribute to the overall survival of newly diagnosed patients with GBM. Even though patients with GBM show poor preoperative functional status, a structured early inpatient rehabilitation intervention may be improved functional outcomes after surgery.

## Figures and Tables

**Table 1:** Patient’s clinical and tumor characteristics.

	Overall (*n* = 105)	Deterioration Group (*n* = 25)	No Remarkable Change Group (*n* = 55)	Good Recovery Group (*n* = 25)	*p* Value
Age (years)	67.0 ± 14.1	71.0 ± 12.2	66.7 ± 11.5	64.6 ± 11.5	0.24
Men/Women (*n*)	59/46	11/14	31/24	16/9	0.34
KPS at admission (median)	57.9 ± 15.7 (60)	61.2 ± 10.9 (60)	59.6 ± 18.6 (60)	50.4 ± 8.4 (50)	0.01
Extent of resection (*n*)					0.14
Gross and near total resection	65	14	30	21	
Partial resection	23	6	15	2	
Biopsy	17	5	10	2	
Treatments (*n*)					0.10
Surgery only	5	1	4	0	
Surgery + RT or TMZ or Bev	10	4	6	0	
Surgery + RT concomitant TMZ	70	15	33	22	
Surgery + RT concomitant TMZ + Bev	20	5	12	3	
Tumor location (*n*) ^†^					
Frontal	35	10	19	6	0.49
Parietal	23	7	8	8	0.15
Temporal	42	9	22	11	0.85
Occipital	7	3	3	1	0.46
Others	17	5	8	4	0.83
Tumor hemisphere (*n*)					0.16
Right	45	11	24	10	
Left	49	9	25	15	
Bilateral	11	5	6	0	
Tumor size (mm)	44.5 ± 13.8	48.0 ± 14.0	42.6 ± 13.1	50.0 ± 9.8	0.14
Lengths of hospital stay (days)	60.3 ± 16.4	59.2 ± 16.8	62.3 ± 18.7	57.0 ± 8.8	0.40

Values are mean ± SD. KPS, Karnofsky performance status; TMZ, temozolomide; RT, radiation therapy; Bev, Bevacizumab. ^†^ Some patients had a combined tumor location.

**Table 2:** Comparison of the deterioration and good recovery groups based on pre-, intra-, and post-operative factors.

	Deterioration Group	Good Recovery Group	*p* Value	Effect Size
Preoperative factors				
Length from initial symptoms to surgery (days)	67.3 ± 45.8	37.8 ± 19.9	0.01	0.83
Initial Symptoms (*n*) ^†^				
Motor paralysis and muscle weakness	5	13	0.03	0.34
Cognitive dysfunction	14	10	0.25	0.23
Headache	3	3	1.00	0.19
Visual field defect	3	1	0.60	0.00
Dysphagia	0	1	1.00	0.14
Fatigue	0	1	1.00	0.14
Intraoperative factors				
Surgery time (minutes)	555.0 ± 133.9	526.9 ± 127.7	0.47	0.22
Bleeding volume (mL)	692.0 ± 588.3	644.3 ± 529.3	0.78	0.09
Transfusion volume (mL)	117.9 ± 234.5	73.0 ± 210.5	0.51	0.20
Infusion volume (mL)	4086.0 ± 1144.5	4096.0 ±1108.5	0.98	0.01
Fluid balance (mL)	1317.0 ± 771.3	796.3 ± 929.9	0.05	0.60
Postoperative factors ^#^				
Fever (*n*)	6	4	0.73	0.10
Infection (*n*)	4	2	0.67	0.12
Pneumonia (*n*)	1	0	1.00	0.14
Cerebral hemorrhage (*n*)	9	4	0.20	0.23
Ischemic stroke (*n*)	5	1	0.10	0.24
Motor paralysis (decreases BRS 2 stage or more) (*n*)	9	1	<0.01	0.43
Length from surgery to chemoradiotherapy (days)	19.1 ± 8.8	17.0 ± 4.3	0.28	0.30
RT and TMZ tolerance (*n*)				
RT interruption or discontinuation	3	1	0.11	0.29
TMZ interruption or discontinuation	8	3	0.14	0.28

Values are mean ± SD. ^†^ Patients with GBM have more than one type of symptoms. ^#^ Patients with GBM have more than one type of symptoms were collected at 7 days after surgery. BRS: Brunnstrom recovery stage; TMZ, temozolomide; RT, radiation therapy; GBM, glioblastoma multiforme.

**Table 3:** Comparison of the deterioration and good recovery groups based on rehabilitation-related factors.

	Deterioration Group	Good Recovery Group	*p* Value	Effect Size
Length up to rehabilitation onset (days)	2.6 ± 1.5	3.4 ± 2.3	0.17	0.39
Length up to sitting training onset (days)	4.8 ± 2.9	5.6 ± 5.1	0.54	0.19
Length up to walking training onset (days)	13.2 ± 16.7	8.4 ± 7.9	0.20	0.37
Motor paralysis of (median of BRS)				
admission	4.9 ± 1.2 (5)	4.4 ± 1.4 (5)	0.26	0.20
after surgery	3.7 ± 1.4 (4)	4.3 ± 1.3 (5)	0.19	0.22
discharge	4.0 ± 1.4 (4)	5.5 ± 0.9 (6)	<0.01	0.55
No motor paralysis (*n*)				
admission	6	9	0.53	0.11
after surgery	2	9	0.04	0.32
discharge	2	10	0.02	0.36
Change from admission to discharge (*n*)				
Deterioration of motor paralysis	13	1	<0.01	0.57
Sever cognitive disorder or depression	10	0	<0.01	0.50

Values are mean ± SD. Motor paralysis showed the Brunnstrom recovery stage (BRS) of the lower limb.

**Table 4:** Hematologic and non-hematologic toxicity during chemoradiotherapy in the deterioration and good recovery groups.

	Deterioration Group			Good Recovery Group			*p* Value	Effect Size
	Grade 0	Grade 1–2	Grade 3–4	Grade 0	Grade 1–2	Grade 3–4		
Hematologic toxicity *n* (%) ^†^								
Leukopenia	10 (45)	9 (41)	3 (14)	15 (60)	9 (36)	1 (4)	0.26	0.29
Neutropenia	18 (78)	2 (9)	3 (13)	16 (67)	4 (17)	4 (17)	0.91	0.14
Lymphocytopenia	4 (17)	11 (48)	8 (35)	7 (29)	14 (58)	3 (13)	0.26	0.33
Thrombocytopenia	3 (13)	20 (87)	0 (0)	5 (20)	20 (80)	0 (0)	0.48	0.17
Anemia	3 (13)	21 (87)	0 (0)	4 (16)	19 (76)	2 (8)	0.18	0.35
Non-hematologic toxicity *n* (%)								
Constipation	9 (39)	14 (61)	0 (0)	14 (56)	10 (40)	1 (4)	0.36	0.26
Fatigue	6 (30)	14 (70)	0 (0)	20 (80)	5 (20)	0 (0)	<0.01	0.50
Fever	11 (46)	13 (54)	0 (0)	22 (88)	3 (12)	0 (0)	<0.01	0.45

^†^ 1 patient missing for neutropenia and lymphocytopenia in the good recovery group and 1–5 patients missing for each toxicity in the deterioration group.

## Data Availability

Available from the corresponding author upon reasonable request.
